# Income and clinical depression versus non-clinical mental health: Same associations or different structures? A dissociation strategy using a national representative random survey based on *EUROHIS (INHIS-2)*

**DOI:** 10.1371/journal.pone.0234234

**Published:** 2020-06-09

**Authors:** Yaron Zelekha, Orly Zelekha

**Affiliations:** 1 Faculty of Business Administration, Ono Academic College, Kiryat Ono, Israel; 2 The Israel Ministry of Health's: Israel Center for Disease Control, Ramat Gan, Israel; Medical University of Vienna, AUSTRIA

## Abstract

We use a unique, large survey on health conditions conducted in Israel (10,331 respondents, 52.8% females, mean age 49.8) to explore whether clinical determined depression and/or anxiety has different associations in compare to non-clinical determined terms such as mental health. We find that absolute income is significantly associated with a decrease in negative mental health but have no significant association with depression and/or anxiety prevalence, whereas relative income is significantly associated with a decrease in depression and/or anxiety prevalence but have no significant association with negative mental health. The two specifications differed also with respect to several important risk factors, including personal attributes such as being of African origin, of a non-Jewish Arab minority or divorced. However, they were similar with general characteristics, such as participation in sport and smoking activities, whether a person has children, age and gender. Our results put forth sever questions about the wide use of non-clinical mental health, happiness and wellbeing as a sole proxy for a person's true wellbeing or utility.

## I. Background

The causes of human mental health, wellbeing and happiness have become a highly studied topic in economic literature over the past 15 years [1, 2, 3, 4, 5, 6]. One obstacle facing the empirical literature is how to define and measure these obscure and overlapping terms. In spite of these challenges, researchers have used non-clinical and perceived measures as proxies for a person's utility [3, 7]. However, the use of non-clinical and perceived terms contradicts the revealed preference framework that dominates the economics. These measures are highly dependent on personality because they are not based on actual behavior and are not objectively determined [3, 8].

The economic literature documents the shortcomings of perception-based data in other fields of empirical economics. For example, during the 1980's and 1990's, inflation expectations were measured mainly through consumer, investor and manager surveys of their personal inflation forecasts. Later literature revealed that surveys do not necessarily document actual behavior as measured by directly driven market expectations (based on a comparison of indexed bonds prices to non-indexed nominal bond prices) and do not fully support the Rational Expectations Hypothesis [9, 10]. In any case, central banks do not rely solely on survey measures, instead mixing several measures, each with its own shortcomings [11].

In psychology, the limits of survey measures have been further recognized [12] on the limited correlation between attitudes and actual behavior. Later works identified preconditions that determine the ways in which attitudes may predict behavior, the nonexistence of exogenous affects, in particular; the attitudes must relate to specific and focused behavior and the attitude must be very strong [13, 14, 15]. These preconditions hardly exist in the survey measurement of mental health and wellbeing.

Furthermore, self-reporting may contain bias. For example, people often resist and avoid exposing themselves (more so in personalities). Additionally, respondents may understand the research goals, and wish to please the researcher (i.e., the demand effect) [16]. Others may respond negatively, attempting to discern the experimenter's hypotheses or destroy the study’s credibility (i.e., the screw-you effect) [17]. Respondents may also be unaware to the true nature of the measured phenomenon, leading to unreliable reports [18]. Thus, modern psychology prefers to measure actual performance rather than use self-reporting. Moreover, research has found that people change their behavior when they know they are being measured and examined (the Observer Effect). These methodological problems, well considered in phycology, are relevant to the measure of wellbeing, mental health and happiness in economics.

The aim of this research is to contribute to the literature on depression, mental health and well-being by uniquely comparing the possible effect of numerous risk factors, especially relative and absolute income, on clinical determined depression and/or anxiety versus non-clinical mental health evaluations using the same data set of respondents.

It is highly important to examine whether these alternative mental variables are driven by the same risk factors to determine whether it is appropriate to use solely self-reported perceived data, which is more easily obtained. If otherwise, the results would severely question the vast sole usage of non-clinical data. This empirical approach closely relates to researchers, who suggested a need to examine whether the determinants of happiness are also determinants of choice behavior in order to support the hypothesis that happiness is a good measure of utility [3].

## II. Related literature

Numerous studies have investigated the relationship between potential risk factors and individual mood disorders, finding that some risk factors are unique but many are common for co-morbidity with other disorders, mainly anxiety disorders.

Low income has generally been found to be a risk factor for both mood and anxiety disorders [19, 20, 21, 22]. It has been found that individuals with higher household income were less likely to report depressive symptoms [23]. Others have found similar results for Chinese middle aged and elderly population using expenditure as a proxy for income [24]. Low level of education is often similarly linked although there are exceptions [19].

The literature stresses that the theory behind the relationship between income and depression divide broadly into two groups. First, according to the stress theory, personal resources, such as coping style, self-esteem, mastery and locus of control, buffer the impact of stress on depression. Therefore, high income allows better resources to allocate in order to tackle stress [25].

Indeed, causal links were found between mortgage debt and rent-related issues to psychological health [26]. Additionally, some researchers claim that working as itself, regardless of income, is an important component of life, providing financial security, meaning, identity and social engagement [27]. Studies have also found that status inconsistency (defined as the combination of a high level of education and low income) increases the risk of mood and anxiety disorders [28].

Second, according to the strain theory, difference in community characteristics, such as values, social welfare, social cohesion, infrastructure and public health policy, have a significant impact on depression prevalence.

In this line, some researchers argue that income inequality and poverty are fundamental causes of mental disorders because they limit access to resources that promote health [29].

Other researchers claim that socioeconomic status does not play a major role in determining the level of treatment for depression and anxiety disorders. In this regard, poor outcomes for individuals of a low socioeconomic status with depression and anxiety may be connected to differences in the quality of care rather than the level of care [30]. In addition, several studies indicate that low socioeconomic status is correlated to other potential risk factors for depression, including physical activity [31].

Alternatively, several other papers found evidence that relative income matters to self-reported work satisfaction [32, 33]. Others have expanded the potential reference group to which individuals are compared found empirical support for local, regional and national income comparisons or inequality [34, 35, 36, 37, 38, 39]. Some even found that increased relative income inequality (in compare to county's level of income) is positively correlated with U.S. suicide risk, which was used as a proxy for happiness [40]. Other research have found that youth who perceive their relative household income to be low also report high-perceived stress, depression and worse general health [41].

Non-economic risk factors for mood disorders that have been identified include young age, being unmarried, a higher self-rated stress level, poorer self-rated mental health, dissatisfaction with life, gender (female), not being fully employed (beyond the income implications), self-rated medical conditions, race and ethnicity. Moreover, there is a direct relationship between the number of risk factors a person has and their probability of having a mood disorder. It seems that as the number of risk factors increases, the probability of having a mood disorder also increases [27, 30]. Other studies have found that health ailments and co-morbid depression have worse medical outcomes [e.g., for asthma, 23; for chronic obstructive pulmonary disease, 42]. Indeed, it has been found that even depressive symptoms (as opposed to a depression diagnosis) were associated with higher mortality rates [42].

Our research correlates with the above mentioned literature by examining the role of both absolute as well as relative income on depression and on mental health. However, it also differs from the literature in important aspect, by examining the role of income on both clinical and non-clinical measures takes from the same sample of respondents. As far as we know, this is the first research who use this dissociation strategy in order to asses whither the sole usage of either one of them in research is reliable.

## III. Methods

### Sample

The Israeli Ministry of Health's Center for Disease Control (ICDC) conducted the second INHIS-2 during 2007–2010. INHIS-2 was based on the European Health Interview Survey (EUROHIS) framework initiated in 2000 by the World Health Organization’s Regional Office for Europe. Informed consent for each participant was obtained by telephone after a brief explanation about the health survey, including the objectives and the survey's importance. People who consented by telephone to participate in the survey were told that they could stop the interview at any point and could decline to answer any question at any time. According to Israeli legislation, such a telephone health survey, which is conducted within the capacity of the ICDC as a regulator, does not require an approval of an ethical committee. Therefore, no such approval was needed for this data collection and analysis. Telephonic consent to be interviewed and the fact that the interview can be stopped at any time according to the interviewee's wish assure that all participants agreed to take part in the survey.

The INHIS-2 was organized into four modules (women's health, use of health services, mental health and health behaviors for prevention). A representative sample of the general population of Israeli citizens aged 21 years and over was calculated using nQuery software (https://www.statsols.com/nquery), which is a common clinical tool for sample size calculation. The sample size was calculated based on the known rate of smokers in the Israeli general population at the time because of the known association of smoking with comorbidity and especially with depression [43, 44]. The sample size calculation was 2,359 participants (Two sided interval test, Expected proportion of current smokers based on previous surveys 25.1%, 95% Confidence level, 1.75% Distance from proportion to limit). In fact, four representative samples were collected, each to any of the four modules (therefore actual distance from proportion to limit was well below 1%). Since most of the questionnaires were identical including health ailment such as depression and/or anxiety (besides the questions that belonged to the specific module) than for most of the variables the sample size was four times the required sample.

Telephone numbers were randomly selected from a computerized list of national telephone company subscribers. According to the Israeli Central Bureau of Statistics [45], 94% of Israeli households had telephone lines. Of 29,697 eligible households, 11,906 households (42.1%) could not be located. Of 17,791 contacted households, there was a 58.1% response rate, 10,331 men and women completing the full questionnaire (among them 3,006 completed the mental health module). Of those, 77.8% were Israeli Jews and 22.2% were Israeli Arabs; 47.2% were male and 52.8% were female.

Non-responses included outright refusals, partially completed interviews, non-located individuals and repeated postponements. Informed consent for each participant was obtained over the telephone. The 150 EUROHIS questions were translated into Hebrew and back into English to ensure correct translation. INHIS-2 included 142 questions after eight questions were deemed irrelevant to Israelis and removed. An additional 129 questions relevant to Israelis (regarding population group, etc.) or related to specific topics of interest were added to INHIS-2. The Hebrew questionnaire was translated into Arabic and Russian, translated back to Hebrew for quality control. Each module was pilot-tested and contained the same core of 86 questions plus an additional set of 66–79 questions that addressed a specific topic. The questionnaire was administered over the telephone by trained interviewers from the corresponding population group in Hebrew, Arabic or Russian.

### Measures

In INHIS-2, respondents were classified as having a health ailment if they reported yes for the following items: (a): Do you have or have you ever had (index condition)? (b): Was this condition diagnosed by a doctor? (c): Was this condition present in the previous 12 months? (d): Did you take medications or receive any form of treatment for the condition during the 12 months prior to the interview? We included respondents as having a depression and/or anxiety only respondents that answered yes to all of these questions. That is, respondents that were diagnosed by a doctor for depression and/or anxiety during the past 12 months and also were given by the doctor with medications or treatments.

In one of the four modules (mental health, see sample section) respondents were asked to rank also their mental health, physical health and general health on a scale of 1to 5 (1 for very good and 5 for very bad). We have used the data for mental health and defined the answers of bad or very bad (4 for bad and 5 for very bad) in a categorical variable as indicating negative mental health (compared to the answers 3 for average, 2 for good and 1 for very good, all defined as indicators of positive mental health). 4.06% of the sample was categorized as people with a negative mental health, compared to 4.30% identified as having depression and/or anxiety. In addition, we also examined all the 5 categories data for mental health, without dichotomized the data into negative or positive mental health.

Income was defined in three levels that is below-mean net income for a household (up to 8499 NIS), mean net income for a household (between 8500–9000 NIS) and above-mean net income for a household (more than 9001 NIS). The range of 8500–9000 NIS for a household is the mean household income in Israel at the time. Unfortunately, we were not able to collect specific income data because of people usually are reluctant to provide full income measures. Therefore, we had to settle for an income report that is between margins.

We used a logistic analysis that incorporates both continuous variables and categorical variables. Therefore, we were able to use the income levels as a categorical proxy measure for absolute income. The inclusion, of levels of income in a categorical manner express how changes in the level of absolute in compare to other levels of income correlates with difference in the dependent variables. In this sense, our measure for absolute income also measures relative income, however relative to other levels of income and not relative to peers' income.

In parallel, we used the income levels as a parameter in measures for relative income. i.e., measures, which are based on a difference between each respondent's levels of income in compare to peers levels of income.

Following the literature, we define relative income in comparison to "people like me" and uniquely examined three measures of relative income simultaneously, which allows us to differentiate between social and inner comparisons [3]. The first measure of relative income, socioeconomic relative income, is the difference between absolute income level and the level of socioeconomic group clusters of the individual's municipality. This measure has wide use in the literature [8]. The socioeconomic (SE) cluster of the place of residence was assigned by the Israel Central Bureau of Statistics. Ranks were on a scale of 1 to 10, and a lower rank indicates a lower SE status. This SE index reflects a combination of basic characteristics, such as the financial resources of the municipality, housing density, the proportion of residents who own a vehicle, and the education and employment profile of the residents [45]. We divided the ten categories of socioeconomic clusters into three groups (low level of socioeconomic clusters were ranks of 1 to 4, medium level of socioeconomic clusters 5 to 7 and high level of socioeconomic clusters 8 to 10) and indexed them (1 for the low socioeconomic clusters, 2 for the medium socioeconomic clusters and 3 for the high socioeconomic clusters). We also indexed whether an individual’s income was below mean, mean and above mean in the same manner, then calculating the difference between a person’s income index and socioeconomic cluster index. The second measure of relative income, education relative income, is the difference between absolute income level and the individual's level of education (because education can serve as a proxy for both income expectations and self-esteem). We categorized years of education into three categories (low level was 1 to 11 years of education, medium level was 12 to 15 years and high level was 16 years and above), indexed them in the same manner and calculated the difference between absolute income's levels and education levels. The third measure of relative income, socioeconomic education relative income, is the difference between the socioeconomics index and the educations index. In addition, economic theory often dictates that the relevant measure of welfare is consumption and not income. Some researchers claimed that net worth may be a better proxy for current consumption than is transitory income [46]. Therefore, we included an alternative measure based on the number of rooms per living capita. It should be mentioned that, in the smaller mental health sample, we had to group the relative income variables into two categories of positive or no difference and negative difference, whereas we could arrange five categories in the larger depression and/or anxiety sample (no difference and two levels of positive and negative differences).

### Statistical analyses

The univariate logistic analysis included a t-test for continuous variables and a chi-squared test for categorical variables. Statistically significant variables in the univariate analysis were then included in the multivariate models (after ruling out potential multicollinearity). The odds ratios (OR) and the 95% confidence intervals (95%CIs) were derived from these models, controlling for confounding variables. Statistical analyses were conducted using SAS 9.1. All data descriptions, sources and statistics are given in Table 1.

**Table 1:** Summary statistics.

	All sample		Only mental health module was included		P Value**	Definitions
Variable	N (%) N = 10,331	Depressed and/or Anxious Individuals N (%) N = 444 (4.30%)	All sample N (%) N = 3006	Negative wellbeing Individuals N (%) N = 122 (4.06%)		
Income:						
Below-mean Income	3960 (43.39)`	265 (64.17) *	1090 (40.48)	96 (96.50) ^+^	<0.0001	Up to 8499 Nis
Mean Income	2341 (25.65)	86 (20.82)	754 (28.00)	11 (9.91)		Between 8500–9000 NIS
Above-mean Income	2825 (30.96)	62 (15.01)	849 (31.53)	4 (3.60)		More than 9001 NIS
Socioeconomic relative income:						Differences in category of income in comparison to the category of socioeconomic cluster
No difference:	492 (42.2)	127 (33.3)*	1102 (44.6)	60 (55.6) ^+^	0.0007	
1 level positive difference:	1973 (23.9)	50 (13.1)	643 (26.0)	6 (5.6)		
2 level positive difference:	554 (6.7)	8 (2.1)	174 (7.0)	2 (1.9)		
1 level negative difference:	1852 (22.4)	156 (40.8)	471 (19.1)	33 (30.6)		
2 level negative difference:	398 (4.8)	41 (10.7)	80 (3.2)	7 (6.5)		
Education relative income						Differences in category of income in comparison to the category of years of education***
No difference:	4220 (46.3)	185 (44.9)*	1194 (43.6)	53 (44.5) ^+^	<0.0001	
1 level positive difference:	1607 (17.6)	54 (13.1)	342 (30.8)	43 (36.1)		
2 level positive difference:	142 (1.6)	5 (1.2)	261 (9.5)	6 (5.0)		
1 level negative difference:	2506 (27.5)	133 (32.3)	380 (13.9)	11 (9.2)		
2 level negative difference:	645 (7.1)	35 (8.5)	61 (2.2)	6 (5.0)		
Socioeconomic education relative income						Differences in category of education in comparison to the category of socioeconomic cluster****
No difference:	4009 (43.01%)	185 (44.79%)*	1210 (45.0)	44 (39.6) ^+^	<0.0001	
1 level positive difference:	2717 (29.15%)	91 (22.03%)	508 (18.9)	5 (4.5)		
2 level positive difference:	824 (8.84%)	11 (2.66%)	50 (1.9)	1 (0.9)		
1 level negative difference:	1512(16.22%)	96 (23.24%)	734 (27.3)	49 (44.1)		
2 level negative difference:	260 (2.79%)	30 (7.26%)	187 (7.0)	12 (10.8		
Education (Mean ± SD)	13.53 ± 4.03	12.47±3.78*	13.7±3.94	11.9±4.2^+^	0.08	Years of education
Cluster 5–7	4260 (41.24%)	236 (52.8)*	1180 (39.3)	47 (38.5)	<0.0001	Individuals who live in municipalities ranked 5–7 according to the central bureau of statistics
Cluster 8–10	1664 (16.11%)	77 (17.2)*	440 (14.6)	9 (7.4) ^+^	0.25	Individuals who live in municipalities ranked 8–10 according to the central bureau of statistics
Non Jewish Arab minority	2298 (22.24%)	63 (14.1)*	828 (27.5)	61 (50) ^+^	<0.0001	Individuals from the Arab minority
Israeli born	4592 (44.45%)	121 (27.07%)*	3006 (100)	122 (100)	<0.0001	Individuals which were born in Israel and not immigrated from other country
Asia and African origin	1424 (13.78%)	97 (21.70%)*	396 (13.2)	8 (6.6) ^+^	0.9	Individuals which their fathers were born in Asia and Africa
Sport	5230 (50.80%)	179 (40.22%)*	1626 (54.6)	37(30.8) ^+^	<0.0001	Individuals who frequently engage with 20 minutes of sport activity
Gender	4880 (47.24%)	170 (3.48%)*	1508 (50.2)	53 (43.4)	<0.0001	Males
Age (Mean ± SD)	49.76 ± 16.24	56.19±15.73*	49.1±15.9	52.6±16.4^+^	0.02	Individuals' age in years
Child (Mean ± SD)	2.76 ± 2.01	2.54±1.79*	2.8+2.0	2.8+2.1	0.3	Individual's number of children
Smoke	2357 (22.81%)	123 (27.52%)*	696 (23.2)	35 (28.7)	0.001	Individuals which currently smokes
Employee	662 (6.41%)	82 (18.34%)*	189 (6.2)	28 (23.0) ^+^	<0.0001	Unemployed or disabled individuals
Divorced	635 (6.15%)	71 (15.88%)*	163 (5.4)	14 (11.4) ^+^	0.9	Individuals who divorced their spouse
Widowed	655 (6.44%)	56 (12.53%)*	128 (4.6)	11 (9.0) ^+^	0.96	Individuals who lost their spouse
Single	1061 (10.27%)	38 (8.50%)*	295 (9.8)	11 (9.0)	0.009	Unmarried individuals

*Significant difference between depressed individuals and not depressed individuals

+ Significant difference between individuals that reported negative wellbeing and individuals with better wellbeing reports

** Significant difference between depressed individuals and individuals with negative well being

*** Education relative income is the difference between absolute income level and the individual's level of education. We categorized years of education into three categories (low level was 1 to 11 years of education, medium level was 12 to 15 years and high level was 16 years and above) and calculated the difference between absolute income's levels and education levels. Positive difference means that the level of income is larger than the education level.

**** Socioeconomic education relative income is the difference between the socioeconomics level and the educations level. Positive difference means that the socioeconomics level is larger than the education level.

## IV. Results

As can be seen in Table 1 and in Table 2, there are no major differences between the Israel population data and the sample data. There are some under representation of the younger ages and households living in low level of socioeconomic clusters. However, these small differences are not extreme and can only work to undermine any possible negative correlation between income and depression. That is, if any correlation is to be found in the sample it is probable even more significant it the general population. In any case, when adjusting the data for these differences, our results have not been significantly altered.

**Table 2:** Demographic characteristics of the sample and the Israeli population.

Variable	All Sample	Israel Population
Income*	43.4%	46.6%
Education**	13.5	13.4
Cluster 5–7***	41.2%	47.2%
Cluster 8–10***	16.1%	16.7%
Non Jewish Arab minority****	22.2%	24.4%
Israeli born*****	44.4%	46.9%
Asia and African origin	13.8%	13.5%
Gender	47.2%	48.3%
Age	49.8	47.5
Child	2.8	2.9
Employee******	6.4%	6.7%
Divorced*******	6.2%	7.7%
Widowed*******	6.4%	6.7%
Single*******	10.3%	13.1%

* Net income for a household up to 8,500 NIS, which was defined in our sample as below mean income. Most of the difference is probably a result of the availability of the Israel population data for the ages 18+ compare to the sample data for the ages 21+

** Average years of education. Some of the difference is probably a result of the unavailability of the Israel population data for 2009 but rather of 2015

*** Some of the difference may be a result of the availability of the Israel population data for the year 2008 while the sample was collected at 2007–2010

**** Most of the difference is probably a result of difference in definitions. The Israel Central Bureau of Statistics report non Jews married to Jews as non Jews while the Israeli public consider them as part of the Jewish environment

***** Most of the difference is probably a result of the in availability of the Israel population data for the ages 21+ which required calculation based on the data for the total Jewish Israel data adjusted for both non Jewish Arab minority as well as for the ages 21+

****** Most of the difference is probably a result of the difference in age characteristics of the data. The sample data comprised of people age 21+ while the Israel population data for the unemployed parameter is according to accepted definition of unemployed people

******* Most of the difference is probably a result of the difference in age characteristics of the data. The sample data comprised of people age 21+ while the Israel population data for the Divorced, Widowed and Single parameters is of people age 25+

Furthermore, our dissociation strategy also allows us to mitigate the risk of sample selection bias. Since, there is no reason to assume that the possible dissociation of absolute and relative income effects (between the depression and/or anxiety specification and the mental health specification) is dependent upon sample characteristics. That is, for example, even if the sample is unknowingly not representative in a certain age cohort, the unrepresentative characteristic will be similar in both depression and/or anxiety as well as negative mental health samples. Therefore, if a dissociation of effects will be identified between the two samples, the difference will not be a result of the unrepresentative characteristic.

We used two comparable specifications that share numerous risk factors identified by the literature as being associated with depression or negative mental health. The specifications differed in their dependent variable—depression and/or anxiety versus negative mental health—and shared a large set of controlled risk factors. Both specifications had a relatively high explanatory power. Moreover, the estimations were stable and robust to the numerous controls, minimizing the risk of omitted variables.

Whereas absolute income had an insignificant effect on depression and/or anxiety with (see Model B-3 versus Model B-5, Table 4), it had a robust significant effect on negative mental health [OR = 0.25, 95%CI (0.13–0.47] (see Model A-5, Table 3). Moreover, the insignificance of absolute income on depression and/or anxiety was robust to alternative specifications or different sampling of health conditions, numerous control variables and several sub samples divided by gender, age and religion. It should be stressed that the absolute income variable remained significant in the presence of the socioeconomic education relative income variable. However, when incorporating both socioeconomic relative income and the socioeconomic education relative income variables into that specification, the absolute income variable lost its significance (see Model B-4 versus Model B-5, Table 4).

**Table 3:** Factors associated with negative wellbeing.

Variable	Model A-1 OR (95%CI)	Model A-2 OR (95%CI)	Model A-3 OR (95%CI)	Model A-4 OR (95%CI)	Model A-5 OR (95%CI)	Model A-6 OR (95%CI)
Number of observations	2947	2437	.2672	2672	2437	2437
Income	0.20++ (0.11–0.36)	-	-	0.19++ (0.10–0.36)	0.25++ (0.13–0.47)	0.25++ (0.13–0.48)
Socioeconomic relative income	-	0.26++ (0.14–0.48)	-	-	0.54 (0.27–1.08)	0.54 (0.27–1.09)
Education relative income	-	-	0.43++ (0.29–0.66)	0.82 (0.52–1.3182)	-	-
Socioeconomic education relative income	-	-	-	-	-	-
Education	0.98 (0.93–1.04)	0.95 (0.90–1.01)	-	-	0.98 (0.92–1.03)	0.98 (0.92–1.03)
Cluster 5–7	2.39 (1.19–4.82)	-	-	-	-	-
Cluster 8–10	1.31 (0.51–3.36)	-	-	-	-	-
Non Jewish Arab minority	5.39++ (2.56–11.38)	7.25++ (3.72–14.13)	5.10++ (3.01–8.65)	3.28++ (1.87–5.75)	4.23++ (2.03–8.82)	4.16++ (2.00–8.70)
Asia and African origin	0.71 (0.31–1.58)	0.69 (0.29–1.62)	0.98 (0.44–2.17)	0.87 (0.39–1.94)	0.71 (0.30–1.6879)	0.72 (0.30–1.70)
Sport	0.50++ (0.34–0.76)	0.46++ (0.30–0.73)	0.43++ (0.28–0.66)	0.49++ (0.32–0.76)	0.50++ (0.32–0.79)	0.51++ (0.32–0.80)
Gender	0.66 (0.43–1.02)	0.61+ (0.39–0.98)	0.56++ (0.36–0.88)	0.60+ (0.378–0.94)	0.61+ (0.38–0.98)	0.61+ (0.38–0.97)
Age	1.02++ (1.01–1.04)	1.02+ (1.00–1.04)	1.04++ (1.02–1.05)	1.03++ (1.01–1.04)	1.02+ (1.00–1.04)	1.02+ (1.00–1.04)
Child	0.87+ (0.77–0.98)	0.87+ (0.76–0.99)	0.91 (0.80–1.02)	0.88+ (0.77–0.99)	0.86+ (0.75–0.98)	0.85+ (0.74–0.97)
Smoke	1.30 (0.82–2.05)	1.30 (0.80–2.11)	1.46 (0.91–2.33)	1.37 (0.86–2.20)	1.33 (0.82–2.17)	1.33 (0.82–2.16)
Employee	4.11++ (2.46–6.86)	4.26++ (2.46–7.38)	4.95++ (2.91–8.41)	3.69++ (2.15–6.34)	3.66++ (2.10–6.34)	3.68++ (2.11–6.40)
Divorced	1.86 (0.93–3.72)	1.64 (0.76–3.54)	1.2 (0.86)	1.40 (0.66–2.99)	1.37 (0.64–2.97)	1.42 (0.65–3.11)
Widowed	1.61 (0.76–3.41)	1.49 (0.66–3.38)	1.62 (0.74–3.52)	1.32 (0.60–2.87)	1.29 (0.57–2.91)	1.35 (0.59–3.11)
Single	0.73 (0.32–1.63)	0.92 (0.41–2.09)	1.33 (0.59–3.00)	1.08 (0.48–2.42)	0.93 (0.41–2.10)	0.95 (0.42–2.18)
Rooms Per Capita	-	-	-	-	-	0.91 (0.64–1.28)
C	0.84	0.80	0.81	0.84	0.83	0.83

* The dependent variable in all Models is non-clinical subjectively defined negative wellbeing.

+ Significant at the 5 percent level.

++ Significant at the 1 percent level.

**Table 4:** Factors associated with depression and/or anxiety.

**Variable**	Model B-1 OR (95%CI)	Model B-2 OR (95%CI)	Model B-3 OR (95%CI)	Model B-4 OR (95%CI)	Model B-5 OR (95%CI)	Model B-6 OR (95%CI)	Model B-7 OR (95%CI)
Number of observations	10160	8205	8205	9289	8244	8175	3810
Income	0.73++ (0.58–0.92)	-	0.82 (0.59–1.15)	0.72++ (0.57–0.91)	-	-	0.86 (0.58–1.28)
Socioeconomic relative income	-	0.75++ (0.66–0.86)	0.80++ (0.67–0.96)	-	0.79++ (0.68–0.92)	0.80++ (0.69–0.93)	0.79+ (0.64–0.98)
Education relative income	-	-	-	-			-
Socioeconomic education relative income	-	-	-	0.80++ (0.71–0.90)	0.87+ (0.75–1.00)	0.87 (0.75–1.01)	-
Education	0.96++ (0.94–0.99)	0.96+ (0.93–0.99)	0.96+ (0.94–0.99)	-	-	-	0.97 (0.94–1.00)
Cluster 5–7	1.30 (0.98–1.71)	-	-	-	-	-	-
Cluster 8–10	1.30 (0.93–1.83)	-	-	-	-	-	-
Non Jewish Arab minority	0.94 (0.64–1.37)	0.98 (0.69–1.41)	0.90 (0.61–1.33)	0.96 (0.68–1.36)	1.16 (0.82–1.64)	1.11 (0.78–1.59)	1.06 (0.66–1.69)
Israeli born	0.58++ (0.46–0.74)	0.56++ (0.43–0.72)	0.56++ (0.44–0.73)	0.57++ (0.45–0.73)	0.55++ (0.43–0.71)	0.55++ (0.43–0.72)	0.57++ (0.41–0.79)
Asia and African origin	1.59++ (1.23–2.07)	1.67++ (1.27–2.21)	1.66++ (1.26–2.20)	1.66++ (1.28–2.17)	1.73++ (1.31–2.28)	1.75++ (1.32–2.30)	1.54+ (1.09–2.19)
Sport	0.72++ (0.59–0.88)	0.70++ (0.56–0.88)	0.71++ (0.57–0.89)	0.72++ (0.58–0.89)	0.69++ (0.55–0.85)	0.69++ (0.56–0.87)	0.67++ (0.52–0.87)
Gender	0.62++ (0.50–0.77)	0.63++ (0.50–0.80)	0.63++ (0.50–0.80)	0.59++ (0.47–0.74)	0.62++ (0.49–0.78)	0.62++ (0.49–0.78)	0.62++ (0.46–0.82)
Age	1.02++ (1.01–1.03)	1.02++ (1.01–1.03)	1.02++ (1.01–1.03)	1.02++ (1.01–1.03)	1.02++ (1.01–1.03)	1.02++ (1.02–1.03)	1.00 (1.00–1.02)
Child	0.90++ (0.85–0.97)	0.90++ (0.84–0.97)	0.90++ (0.84–0.96)	0.91++ (0.85–0.97)	0.91+ (0.85–0.98)	0.91++ (0.84–0.97)	0.91+ (0.84–0.99)
Smoke	1.30+ (1.03–1.64)	1.21 (0.94–1.57)	1.22 (0.95–1.57)	1.24 (0.97–1.58)	1.22 (0.95–1.57)	1.18 (0.92–1.53)	1.42+ (1.05–1.93)
Employee	3.61++ (2.73–4.78)	3.75++ (2.79–5.04)	3.67++ (2.72–4.95)	3.78++ (2.83–5.04)	3.88++ (2.89–5.22)	3.84++ (2.85–5.17)	3.37++ (2.35–4.82)
Divorced	2.00++ (1.47–2.71)	1.91++ (1.38–2.63)	1.85++ (1.34–2.57)	2.14++ (1.57–2.91)	1.93++ (1.40–2.67)	2.15++ (1.53–3.00)	1.95++ (1.33–2.86)
Widowed	1.17 (0.83–1.65)	1.00 (0.69–1.46)	0.99 (0.68–1.44)	1.12 (0.79–1.60)	1.04 (0.71–1.51)	1.19 (0.81–1.77)	1.07 (0.72–1.60)
Single	1.01 (0.66–1.55)	1.09 (0.69–1.71)	1.07 (0.68–1.69)	1.13 (0.74–1.75)	1.16 (0.74–1.81)	1.26 (0.80–2.00)	1.21 (0.67–2.20)
Rooms Per Capita	-	-	-	-	-	0.86+ (0.73–1.00)	
C	0.74	0.75	0.75	0.75	0.75	0.75	0.72

* The dependent variable in all Models is clinical objectively observed depression and/or anxiety

+ Significant at the 5 percent level.

++ Significant at the 1 percent level

In contrast, relative socioeconomic income had a significant association on depression and/or anxiety for both the general sample [OR = 0.79, 95%CI (0.68–0.92] (see Model B-5, Table 4) and a chronic health ailments sub sample [OR = 0.79, 95%CI (0.64–0.98] (see Model B-7, Table 4), and insignificant effect on negative mental health. In addition, the socioeconomic education relative income variable was also found significant for the depression and/or anxiety specification [OR = 0.87, 95%CI (0.75–1.00] (see Model B-5, Table 4), while insignificant for the negative mental health specification. The effect of education relative income was found to be insignificant in all models of the depression and/or anxiety specification (see for example Model B-5, Table 4) and significant only in some models of the negative mental health specification [OR = 0.43, 95%CI (0.29–0.66] (see Model A-3 versus Model A-4, Table 3).

In order to examine the stability of the results we also examined an OLS specification using all the five categories of mental health, without dichotomized the data into negative or positive mental health. The results were stable assessing that as absolute income increases the mental health significantly deteriorates through all of the categories (b = -0.32, p<0.0001) and not only between the dichotomized positive and negative mental health (while the relative socioeconomic income remained insignificant, p = 0.36).

Based on their odds ratio calculation, it appears that a one-unit decrease of the socioeconomic relative income variable corresponds to an increase in the prevalence of depression and/or anxiety by 21%. While, a one unit decrease of the socioeconomic education relative income variable corresponds to an increase in the prevalence of depression and/or anxiety by 13% (see Model B-5, Table 4).

The alternative measure based on the number of rooms per living capita was found significant only for the depression and/or anxiety specification [OR = 0.86, 95%CI (0.73–1.00] (see Model A-6, Table 3 and Model B-6, Table 4). However, because the value of real estate depends on the socioeconomic cluster of the asset, we adjusted the variable to each cluster and found that the variable was no longer significant.

In addition, we examined whether the relative income effects are constant for both positive and negative comparisons, although the examination was limited (because of sample size) only to the depression and/or anxiety specification. We divided both of the relative income variables into sub variables, capturing different levels of positive and negative differences. In line with the literature [47], we found that only negative socioeconomic relative income levels are significantly associate with depression and/or anxiety, whereas the association of positive socioeconomic relative income levels are not significant. This effect has diminishes with scale. Regarding the socioeconomic education relative income variable, we found an asymmetric effect of only two level differences and that a one level difference is not significant.

### Control variables

To examine the robustness of the results, a broad range of control variables (including ethnicity, employment, education and health controls) were added to the specifications that may be important in the determination of depression and/or anxiety or of negative mental health.

The first control was ethnicity variables. We found that the variable for being Israeli born was negatively significant [OR = 0.55, 95%CI (0.43–0.71] (this could not be examined at the negative mental health specification due to a lack of samples). The variable for people whose fathers were born in Africa (which according to the literature faces discriminatory environment [48]) was positively significant for the prevalence of depression and/or anxiety [OR = 1.73, 95%CI (1.31–2.28] (see Model B-5, Table 4), and was insignificant for prevalence of negative mental health (see Model A-5, Table 3). In addition, the variable for being of the non-Jewish Arab minority was found to be insignificant at the depression and/or anxiety specification, whereas it was positively significant for the prevalence of negative mental health [OR = 4.23, 95%CI (2.03–8.82].

The second control was general health attitudes variables. We found that the variable for sports activity was negatively significant, corresponding to a decreased prevalence of both depression and/or anxiety [OR = 0.69, 95%CI (0.55–0.85] and negative mental health [OR = 0.50, 95%CI (0.32–0.79] (see Model B-5, Table 4 and Model A-5, Table 3). The variable for smoking was positively significant is several but not all specifications for depression and/or anxiety [OR = 1.30, 95%CI (1.03–1.64] while insignificant for negative mental health (see Models B-1 in compare to B-5, Table 4 and Model A-5, Table 3).

The third control was family conditions variables. We found that the variable for divorced status was positively positivity significant only for depression and/or anxiety specification [OR = 1.93, 95%CI (1.40–2.67] (see Model B-5, Table 4), whereas the variables for widowed or single status were insignificant for both specifications. The variable for having children was negatively significant, corresponding to a decreased the prevalence of both depression and/or anxiety [OR = 0.91, 95%CI (0.85–0.98] and negative mental health [OR = 0386, 95%CI (0.75–0.98] (see Model B-5, Table 4 and Model A-5, Table 3).

The fourth control was personal characteristics variables. We found that the variable for gender (male) was negatively significant, corresponding to a decreased prevalence of both depression and/or anxiety [OR = 0.62, 95%CI (0.49–0.78] and negative mental health [OR = 0.61, 95%CI (0.38–0.98]. The variable for age was positively significant, corresponding to an increased prevalence of both depression and/or anxiety [OR = 1.02, 95%CI (1.01–1.03] and negative mental health [OR = 1.02, 95%CI (1.00–1.04] (see Model B-5, Table 4 and Model A-5, Table 3). The variable for being unemployed or disabled was positively significant, corresponding to an increased prevalence of both depression and/or anxiety [OR = 3.88, 95%CI (2.89–5.22] and negative mental health [OR = 3.66, 95%CI (2.10–6.38] (see Model B-5, Table 4 and Model A-5, Table 3). The variable for years of education (which can also serve as an alternative measure for income) was negatively significant in several but not all of the depression and/or anxiety specifications [OR = 0.96, 95%CI (1.03–1.640.94–0.99] (see Model B-3, Table 4) and insignificant in the negative mental health specification. The variables for religious devotion were found to be insignificant for both specifications.

### Possible omitted variables

When interpreting the findings, a possible source of concern is that other unobserved economic or personal features may drive the estimated effect of income on depression and/or anxiety or on negative mental health, at least in part. Despite our attempts to control for observable factors, the estimates reported may still be biased by unobservable factors correlated with relative income. To assess the likelihood that the estimates are biased by unobservable factors, we followed [49] and used a selection of observables to assess the potential bias. Indeed, in Model A-5, the ratio for absolute income was 1.81, and the ratio for the socioeconomic relative income variable 4.35. In Model B-5, the ratio for the socioeconomic relative income variable was 0.88, and the ratio for the socioeconomic education relative income was 13.95. These relatively high values are not significantly altered under any other Model. Therefore, in order to attribute the entire estimate to selection effects, selection on unobservables would have to be at least as closely wide as selection on observables, which constitutes very large set of controls. In our view, these results make it hardly likely that the estimated effect of income is fully driven by unobservables.

## V. Discussion

Economics and psychology literature gives both absolute and relative utility an independent role in explaining economic behaviors or attitudes, from wealth inequality [50], happiness and job satisfaction [3] to consumption behavior [51] and investment and asset pricing decisions [52].

Against this background, we found that absolute income is significantly associates with a decrease in negative mental health but have no significant association with depression and/or anxiety prevalence, whereas relative income is significantly associates with a decrease in depression and/or anxiety prevalence while have no significant association with negative mental health prevalence. Furthermore, the two specifications differed also with regards to several important risk factors, more personal attributes. In this regard, being of Asia and African origin, of a non-Jewish Arab minority or divorced, and they shared other risk factors that related to general characteristics such as sport and smoking activities, whether one has children, age and gender.

The dissociation of all of these important variables between the depression and/or anxiety specification and the mental health specification has important empirical implications. The use of non-clinical measures as direct proxies for utility is highly accepted in the literature [3]. Therefore, it is very important to establish whether clinical determined mental health, like depression, behaves differently than non-clinical determined mental health, wellbeing and happiness (taken from the same people at the same time). Our results suggest that they do differ; creating questions about the wide use of non-clinical terms as sole proxies for a person's true utility.

Although one can generally expect to have a larger probability of negative mental health than depression and/or anxiety. However, Israel is considered a country in which the self-reports of happiness level is relatively high. According to the last world happiness report (published in support of the UN High Level Meeting on happiness and well-being) Israel is ranked 11^th^ in the world for the year 2017. A surprising result when taking into account that Israel's level of poverty reached the highest level in the OECD countries and Israel's level of inequality reached the second highest level, while the country's security threats are significant. In addition, it is exactly the purpose of this research to undermine the solely use of self-reported wellbeing or negative mental health data.

The results also emphasize the importance of social comparison and inner comparison or habituation. On the one hand, a comparative of current absolute income level to education (which can affect both income expectations as well as self-esteem) has no significant association with depression and/or anxiety or with negative mental health. On the other hand, a comparative of current absolute income level to the socioeconomic current environment as well as education level to the socioeconomic current environment have a significant associations with depression and/or anxiety. These results are consistent with the wellbeing and happiness literature documented by [3, 8]. The former found strong evidence for social comparison in the utility function through the existence of a relative income term, and the latter found that unhappy individuals are more sensitive to comparisons, particularly if the comparisons are unfavorable, as expected from the psychology literature.

It should be stressed that the absolute income variable lost its significance in the presence of the socioeconomic relative income variable for the depression and/or anxiety specification. However, it remained significant in the presence of the socioeconomic education relative income variable. These results suggest that the latter variable may also measure aspects of subjective perceptions of income that the former does not measure.

The findings also suggest that assets and to some extent education may not explain depression and/or anxiety or negative mental health, although the specifications differ by absolute (transitory) income. It seems that income and education have an indirect effect on depression and/or anxiety alone (but not on negative mental health), through comparison with households' living environments. Moreover, since both assets and education can be associate with income in the long term, the results may also suggest that (relative) transitory income has larger associations than (relative) permanent income concerning current depression and/or anxiety or mental health. Unfortunately, our dataset does not allow us to examine this line of thinking so we will leave that for a future research.

In this regard, psychology literature has distinguished between "self-enhancement" and "self-improvement" motives. A concern for status implies that individuals prefer a low socioeconomic reference group ("self-enhancement") and therefore increase their socioeconomic income, while a concern to perform better due to higher goals and standards implies that individuals prefer a high socioeconomic reference group ("self-improvement") and therefore decrease their socioeconomic income level at least in the short term. Unfortunately, our dataset does not include measures of self-enhancement or self-improvement motives. Future research may measure these two motives in order to assess the relationship between them and depression and/or anxiety.

Our results may also have important implications for health policy on possible treatments. Because the wellbeing and happiness literature aims to improve quality of life, it can influence psychological treatment while not accounting for the possible different natures of wellbeing and depression. For example, treatment usually cannot immediately and directly influence current absolute income, but it can sometime influence the depressed patients’ decisions about their living environments or alternatively their perceptions regarding reference groups.

### Limitations

Some caveats must be noted when interpreting the empirical results, and these caveats may also lead to future research. First, our study explored relative income in a categorical framework. However, we were able to asses that only negative socioeconomic relative income levels are significantly associate with depression and/or anxiety, whereas the association of positive socioeconomic relative income levels are not significant. Furthermore, this effect has diminishes with scale.

Second, although we used clinical determined depression and/or anxiety we still had to lean on self-reported survey data. We minimize the potential bias of misunderstanding by using respondents' data that confirmed both diagnose by a physician and taking medications or receiving any form of treatment for the condition. Regarding the risk of under report because of stigma, it is probably more correlated with low income and less educated respondents and therefore less likely to bias the results.

Third, it is known that the prevalence of depression diagnosis is quite low [53]. However, this phenomenon can work statistically against variable significance and therefore we can expect the significant correlations that were found may even be stronger in reality.

Fourth, this research uses a cross sectional data and not a longitudinal data and therefore it cannot identify any causal relationship but only make indirect support for a casual claim. Indeed, theory predicts a possible simultaneous relationship between low income and mood disorders in general, depression in particular [23, for the effect of depression on income, 25 and 29 for the effect of income on depression]. On the one hand, low income negatively contributes to self-esteem, feelings of internal efficiency and ability to diversify activities to more fulfilling alternatives [25]. On the other hand, depression can affect the willingness to work, to advance or to achieve [32, 33].

However, we believe that the risk of reverse causality in our specifications was mitigated because of the following five additive measures:

(1) The participants were instructed to report depression that occurred in the past twelve month. It is reasonable to assume that only the minority of the sample is characterized by earlier depression, which continued into the twelve-month period and only a small portion of this minority is characterized by lifetime chronic depression.

(2) The most important source for reverse causality is the possible effect of depression on the willingness to work. Therefore, we controlled for unemployment, which is more likely than salary income to be affected by depression and/or anxiety or negative mental health. In Israel, salary income can't be reduced without being considered a dismissal practice and therefore non dismissed working respondents have very likely established their salary income years before the current depression and/or anxiety or negative mental health occurred.

(3) We examined two alternative measures for income, education and assets (rooms per capita). It is reasonable to assume that both measures were obtained years before the current depression and/or anxiety or negative mental health occurred. Lagging variables and the use of proxies are accepted methods for tackling possible reverse causality.

(4) We examined the depression specification using a sample of respondents suffering from several chronic health ailments. It is very likely that for these respondents, income was established earlier or at least independent of the current depression, thereby mitigating the role of income as a dominant cause of it.

(5) Reverse causality in most cases can (although not always) be identified by an important variable that results from insignificancy or from an unexpected sign. In our study, income as well as education and assets, were found to be significant with the expected sign for both the negative mental health and depression and/or anxiety specifications in single variant specifications and in multi variant specifications. Only the introduction of relative income resulted in the insignificance of income for the depression and/or anxiety specifications.

## Conclusions

We find that absolute income is significantly associated with a decrease in negative mental health but have no significant association with depression and/or anxiety prevalence, whereas relative income is significantly associated with a decrease in depression and/or anxiety prevalence but have no significant association with negative mental health. The two specifications differed also with respect to several important risk factors, especially personal attributes.

Our results put forth sever questions about the wide use in the empirical literature of non-clinical mental health, happiness and wellbeing as a sole proxy for a person's true wellbeing or utility. It is possible that measures which is diagnosed exogenically based on changes in actual behavior (such as depression) is a significantly different proxy for a person's condition than internal, non-clinical and perceived measures (such as mental health). These results suggests that mental health, happiness and wellbeing are complicated structures and use of a sole proxy may not represent them into a full and reliable extent.
